# Experimental Study on Effects of Aging Time on Dry Shrinkage Cracking of Lime Soils

**DOI:** 10.3390/ma15165785

**Published:** 2022-08-22

**Authors:** Jianwei Yue, Ying Chen, Zhenxian Luo, Siyuan Wang, Huicong Su, Huijie Gao, Yuan Li, Peng Li, Can Ma

**Affiliations:** 1 School of Civil Engineering and Architecture, Henan University, Kaifeng 475004, China; 2 School of Civil Engineering, Central South University, Changsha 410075, China; 3 School of Architectural Engineering, Tianjin University, Tianjin 300072, China

**Keywords:** aging time, lime soil, dry shrinkage cracking, mechanical properties, pH, particle size distribution

## Abstract

The effect of aging on the internal mechanism of the dry shrinkage cracking of lime soil was studied from the perspective of macroscopic cracking phenomenon and microscopic composition change, and the reasonable aging time of lime soil was determined. Large numbers of cracks often occur in buildings constructed using lime soil, which impacts sustainable development and building environmental protection. This study explored the influence of aging time on the mechanical properties and shrinkage cracking of lime soil. The influence of aging time was evaluated using a triaxial compression test; using the dry–wet cycle, sieving, pH, and other tests, the influence of aging time on volume crack rate, expansion shrinkage rate, particle size distribution, and pH was analyzed. Scanning electron microscopy and X-ray diffraction experiments were used to analyze changes in the lime soil particle structure for different aging times and the formation of new substances. The results show that as aging time increases, the stress–strain curve of the soil softens significantly, shear strength deteriorates, and cohesion decreases. When the aging time is 6 h, the expansion rate and shrinkage rate at the center of the soil sample are the maximum. The volume fracture and expansion shrinkage rates decrease first, and then plateau with aging time, with the changes remaining stable after 72 h; these rate decreases are positively correlated with the change rate of pH. The formation of Ca(OH)_2_ affects the sample pH, and the changes in pH, Ca(OH)_2_, and CaO tend to be stable. With an increase in aging time, the proportion of particles of a size less than 0.1 mm decreases, and that of particles of size 0.1–0.5 mm increases. After 72 h of aging, the particle size proportion remains unchanged. Reasonable aging time can, thus, reduce the hydration reaction of lime, improve particle agglomeration effects, and reduce the crack development of the soil.

## 1. Introduction

Lime soil is a common building material for ancient buildings, mainly composed of lime, quartz sand, and clay [1,2,3,4,5]. Most of the existing immovable cultural relics, such as city walls, earth sites, and bridges, also use lime soil as the main repair material, which is related to the high strength, good durability, and strong compatibility of lime soil materials [6,7,8,9]. However, affected by the climate, the dry and wet states in nature are always changing, and the soil moisture content may change periodically, thus, affecting the engineering properties of the soil [10]. Taking Kaifeng, Henan Province, as an example, the studied area has concentrated rainfall throughout the year, high rainfall in summer and autumn, and frequent meteorological disasters such as heavy rain and rainstorms. Kaifeng is located in the Central Plains, and belongs to a typical temperate monsoon climate. It is cold and dry in spring and winter, and hot and rainy in summer and autumn. Therefore, the buildings mainly composed of lime soil experience a large effect from these weather changes and disasters [11]. Under the influence of dry and wet natural factors, lime is prone to hydration reaction, resulting in the reduction in the internal moisture of lime soil. During the process of water absorption expansion and water loss contraction, lime soil undergoes expansion and contraction deformation, which damages its integrity. A large number of cracks appear on the surface of lime soil, which has an important impact on the durability and stability of the soil site [12].

In order to improve the properties of lime soil, the “aging” process was studied by a large number of scholars. The aging process is directly related to the hydration and reactivity of lime soil [13,14,15]. Gu studied the influence of aging time, water–cement ratio, digestion temperature, and other factors on the dispersion and activity of lime milk. The results show that the activity and dispersion of calcium hydroxide are better when the initial lime digestion temperature is 84 °C and the water–cement ratio is 5:1 without aging [16]. Giuseppe found that aging time affects the crystal form and size of calcium hydroxide [17]. Wei explored the mechanism of the lime aging process and its application in the field of cultural site protection. They found that with the increase in aging time, the particle size and activity of calcium hydroxide decreases. Calcium hydroxide with particle sizes of 100–200 mesh can improve the compressive strength, surface hardness, and other physical properties of lime soil [18]. Khattab and Hengiqu found that increasing the aging time can reduce the pozzolanic reaction and improve sample durability [19,20]. However, Sweeney studied the effects of aging time on the mechanical properties of lime-improved clay, and found that as the aging time increases, the density and compressive strength of the sample decrease, and that longer aging times reduce the plasticity indexes of the samples [21].

At present, the research mainly focuses on the effect of aging time on the activity of calcium hydroxide and its engineering application, but there is little research on the aging process law under the joint action of lime and soil. After lime and soil are mixed evenly and reacted with water, the internal physical and chemical changes continue, and lime soil aging takes a certain amount of time [22]. Under the condition that other aging conditions remain unchanged, the aging time is too short, the lower the hydration of lime, the higher the reactivity of lime, and the hydration of lime causes the uplift and cracking of lime soil [23]; long aging time and low lime activity affect the mechanical properties of lime soil [24,25]. Therefore, a reasonable aging time plays an important role in engineering. Through unconsolidated, undrained triaxial shear tests, dry–wet cycles, screening, and pH evaluation, the effects of different aging times on the mechanical properties and dry shrinkage cracking of lime soil were studied at a macro scale; the changes in the particle structure and formation of new substances were analyzed by scanning electron microscopy (SEM) and X-ray diffraction (XRD), and the internal causes of microscale lime soil cracking were explored. The purpose of this paper was to reveal the deterioration mechanism of lime soil from different perspectives of macroscopic cracking phenomenon and microstructure change. By analyzing the quantitative relationship between the pH value change, particle size, and the deterioration degree of lime soil, a reasonable aging time was selected.

## 2. Materials and Methods

### 2.1. Materials

**Figure 1:**
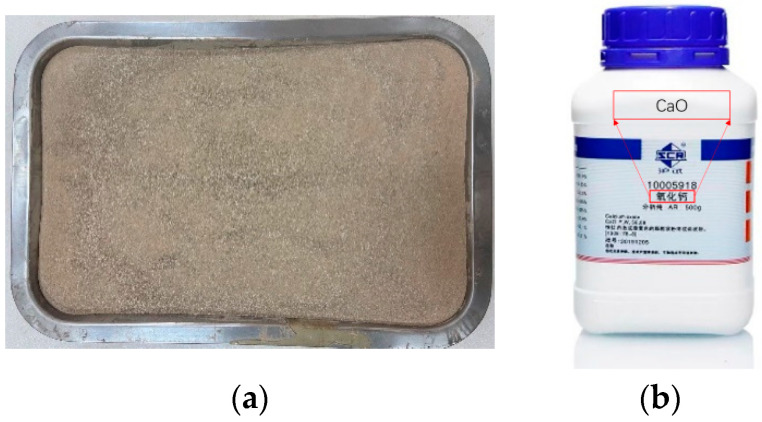
Test materials: (**a**) silty clay in Henan Province; (**b**) quicklime.

The soil used in the test was exfoliated soil from a soil site in Henan Province (Figure 1a). The soil was silty clay, with a liquid limit of 32%, plastic limit of 14%, and plasticity index of 18. The chemical element composition of soil samples was analyzed by XRF test, and the results are shown in Table 1. High active CaO was used for lime soil preparation, with particle size of 150–200 mesh and a white appearance (Figure 1b). The chemical composition of lime is shown in Table 2.

### 2.2. Methods

Seven sets of samples were prepared with aging times of 6, 12, 24, 48, 72, 120, and 168 h, lime content of 20%, and moisture content of 20%. Through unconsolidated, undrained shear tests, dry–wet cycles, screening, pH evaluations, XRD, and SEM, the micro mechanism of the effects of aging time on the dry shrinkage cracking of lime soil were explored experimentally (Figure 1).

**Figure 2:**
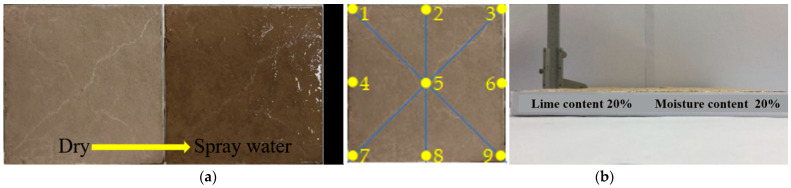
Test process of the samples: (**a**) dry–wet cycle test of sample; (**b**) expansion and shrinkage test of sample.

1. Limit moisture content test: This test used the combined liquid and plastic limit tester to determine the liquid limit and plastic limit of silty clay in Henan Province, calculate the plasticity index, and judge the range of plastic state of silty clay. In this test, 300 g of air-dried soil samples with the optimum moisture content of 27.3% were taken. The moisture content of the prepared soil samples was the plastic limit, the middle value of liquid plasticity, and the liquid limit of the soil, so that the cone penetration depth was controlled at 3~5, 7~9, and 15~17 mm, respectively. They were placed into double-layer, fresh-keeping bags and left for 24 h. The liquid plastic limit was retested. If the penetration depth is not within the specified range, the test shall be reperformed. If the penetration depth is within 3 specified ranges, the test is deemed to be successful, and the liquid limit and plastic limit can be obtained. When the test results met the requirements, the soil samples were placed under the three conditions into the 105 °C oven, and their moisture content was measured to calculate the limit moisture content of silty clay;
2. Aging of soil sample: The soil sample was dried, then the powder was extracted and passed through 2 mm sieve. A total of 3500 g of soil samples was evenly mixed with 700 g of lime, and 840 mL of distilled water was added for uniform mixing. A total of seven groups of samples were prepared. The prepared lime soil sample was placed into the standard oxidation tank (temperature: 20 °C, humidity: 95%), and aged for 6, 12, 24, 48, 72, 120, and 168 h. During the aging process, 30 g samples (3 parallel samples) were taken every 6 h to dry and measure the average moisture content, and water was added to maintain the initial moisture content of 20%;
3. Triaxial compression test: The aged soil samples were proportioned according to the moisture content of 20% and dry density of 1.28 g/cm^3^ to prepare the triaxial samples of diameter 39.1 mm and height 80 mm. The unconsolidated, undrained triaxial shear test was carried out according to the standard for geotechnical test methods (GB/T50123-2019), and the shear rate used was 0.5 mm/min to measure the stress–strain relationship of the sample under confining pressures of 50, 100, and 150 kPa. To reduce the test errors, three parallel tests were conducted in each set of tests with four samples each time, of which three samples were used for triaxial shear under different confining pressures. Since the triaxial test was conducted by computer and acquisition system, the computer automatically recorded the stress value at the corresponding strain, and automatically generated the stress–strain curve and Mohr’s circle after the test started; then the internal friction angle and cohesion of the excavated sample were calculated.
4. Dry–wet cycle test: The dry shrinkage deterioration test was carried out after sample preparation. A total of 2000 g of the aged bulk sample was placed in a mold of size of 25 cm × 25 cm, following which static compaction was performed in a 2 cm steel mold. The moisture content of the sample was 20% and the dry density was 1.28 g/cm^3^, and the static compaction treatment carried out. The lime soil sample was dried in a temperature control oven set at 40 °C for 36 h; after drying, water was sprayed evenly onto the surface of the sample with a spray bottle of 800 mL, which was recorded as a dry–wet cycle (Figure 2a); a total of 5 dry–wet cycles were carried out. During the whole drying process, the sample was weighed by an electronic scale and photographed by a high-definition camera to record the crack development process;
5. Expansion and contraction rate test: A vernier caliper with an accuracy of 0.02 mm was used to measure the height of lime soil samples at 9 different positions, and the average value of the expansion and contraction deformation height of the samples was taken (Figure 2b);
6. Screening test: the aged sample was dried in an oven at 105 °C to constant weight. A total of 200 g dried samples (3 parallel samples) was taken, and a screening test was conducted on the samples with sieve hole diameters of 2, 1, 0.5, 0.25, 0.1, and 0.075 mm. The particle size information of aged lime soil samples was obtained;
7. pH value test: The aged sample was dried, and 100 mL of distilled water was added to 20 g of the dried sample (3 parallel samples), and a pH818 precision pH meter was used to test the pH value of the supernatant after standing;
8. Electron microscope test: The test instrument was a S570 scanning electron microscope. The aged samples were dried for electron microscope test, and the scanning electron microscope multiples were 500 times and 2000 times. The chemical reaction products and microstructure changes in the samples were observed;
9. X–ray diffraction test: An X-ray diffraction test was conducted after the sample was dried. The test instrument was an X-ray diffractometer with model d8-advance, and the scanning incidence angle was 10–70°. The changes in chemical products in the sample were explored.

**Figure 3:**
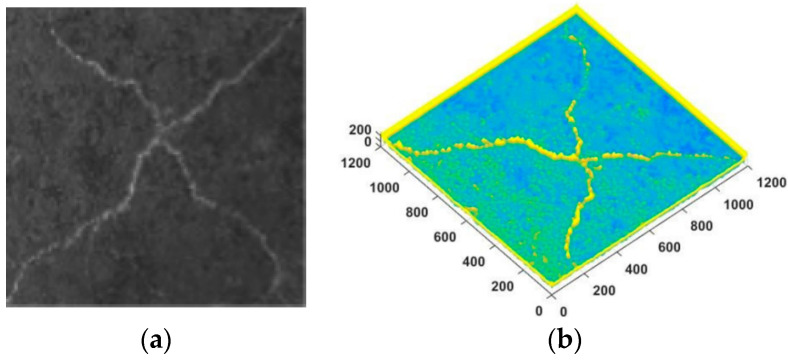
MATLAB 3D image processing: (**a**) specimen crack image; (**b**) 3D image processing.

Crack image processing and information extraction: In order to reduce the influence of sample boundary, the middle part of the sample (size 20 × 20 cm^2^) was used for quantitative fracture analysis. Firstly, Image–pro plus6.0, Adobe PhotoShop, and other software were used to cut the image, correct the size, and remove the lime efflorescence interface. Then, MATLAB software (MATLAB 7.0) was used to compile the program for extracting the characteristic parameters of three-dimensional volumetric fracture rate, and the method of integration was used to solve the volume fracture rate of the sample through the three-dimensional level [26,27]. Figure 3 shows a processed three-dimensional image. Equation (1) is the volume crack rate of soil mass.

(1) $$V_{e}=\overset{N}{\underset{i=1}{\sum }}S\left(M-D_{i}\right)/\left(SMN\right)\;=\sum_{i=1}^{N}\left(255-D_{i}\right)/\left(255N\right)$$

where *S* is the area of each pixel. *S* = 1, *M* is the maximum gray value of the image; *M* = 255, the gray level corresponding to the ith pixel of *D_i_*; and *N* is the total number of image pixels.

## 3. Results and Discussion

### 3.1. Effect of Aging Time on Mechanical Properties of Samples

#### 3.1.1. Stress–Strain Curve

**Figure 4:**
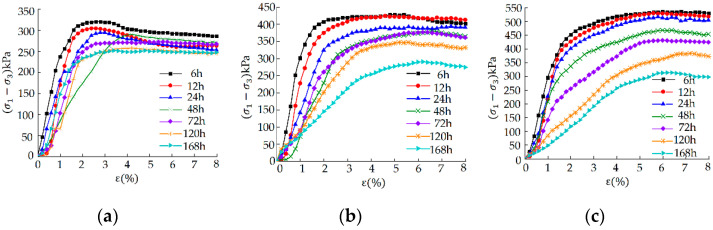
Stress–strain curves of samples under different aging time and confining pressures: (**a**) $\sigma_{3}$ = 50 kPa; (**b**) $\sigma_{3}$ = 100 kPa; (**c**) $\sigma_{3}$ = 150 kPa.

Figure 4 shows the stress–strain curve of the sample under different aging times and confining pressures. When the aging time is 6 h, the stress–strain curve shows strain softening, and the peak stress decreases significantly with an increase in aging time. Under the same confining pressure, for a longer aging time, the peak stress softening amplitude of the curve is greater, and a smaller partial stress is required to produce the same axial strain. There is significant deterioration of the macro shear strength of the soil, but the evolution trends under various confining pressures are different. The decrease in shear strength increases significantly with the increase in confining pressure. When the confining pressure is high, the non-linear characteristics of the shear strength evolution curve are more significant. However, under all confining pressures, the reduction in shear strength is greater for the samples aged for 72–120 h. It is seen from Figure 4c that when the sample is aged for 72–168 h, the shear strength decreases by 37.5% (compared with the ratio of difference with the 72 h result to the value at 72 h).

The reason for the above phenomenon is that as the aging time increases, the lime is in fuller contact with the clay, and obvious agglomeration effects occur. The agglomerates and clay fine particles are wrapped by calcified crystals and silicates to form a hard shell [28]. After compaction, it is difficult to achieve cementation between the clay particles and aggregates, thus, resulting in a reduction in the sample density and mechanical properties [29]. The aging time is a tradeoff at the expense of the mechanical strength of the soil. The longer the aging time, the worse the mechanical strength of the lime soil.

#### 3.1.2. Strength Index Impact Analysis

**Figure 5:**
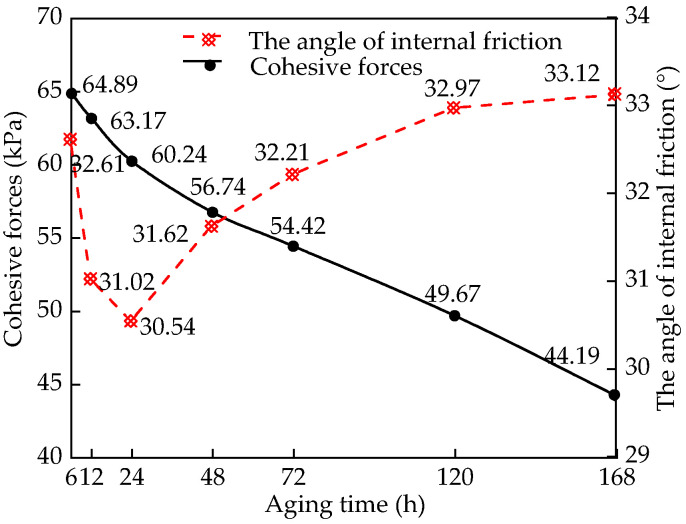
Variation law of cohesion and internal friction angle of samples with different aging time.

Figure 5 shows the relationship between cohesion, internal friction angle, and aging time. The cohesion of the sample increases with an increase in aging time; this is mainly attributed to the carbonation of lime, which forms a layer of calcium carbonate protective shell on the surfaces of the clay particles, resulting in the loss in partial cohesion between the particles. The internal friction angle first decreases, and then increases with an increase in aging time. Owing to short aging time and uneven heat distribution in the soil, the morphology of Ca(OH)_2_ in the hydration reaction is disordered, and the bite friction between the carbonized particles is reduced [30]. With the extension of aging time, the soil particles undergo obvious agglomeration, and the internal friction angle of the lime soil is improved.

The mechanical properties of lime deteriorate with the increase in aging time. The main reason for this is that after the carbonization reaction, the particles only form a carbonization layer on the surface of the soil particles, and the soil particles are only aggregates of loose particles, which cannot achieve strong cementation [31]. Moreover, owing to the carbonization effects, the cohesion between the clay particles is partially lost, resulting in the increase in the internal friction angle and decrease in the cohesion of the remolded lime soil, which affects the other mechanical properties of the lime soil [32]. Therefore, choosing a reasonable aging time is of great significance in engineering applications.

### 3.2. Effects of Aging Time on Sample Volume Crack Rate

**Figure 6:**
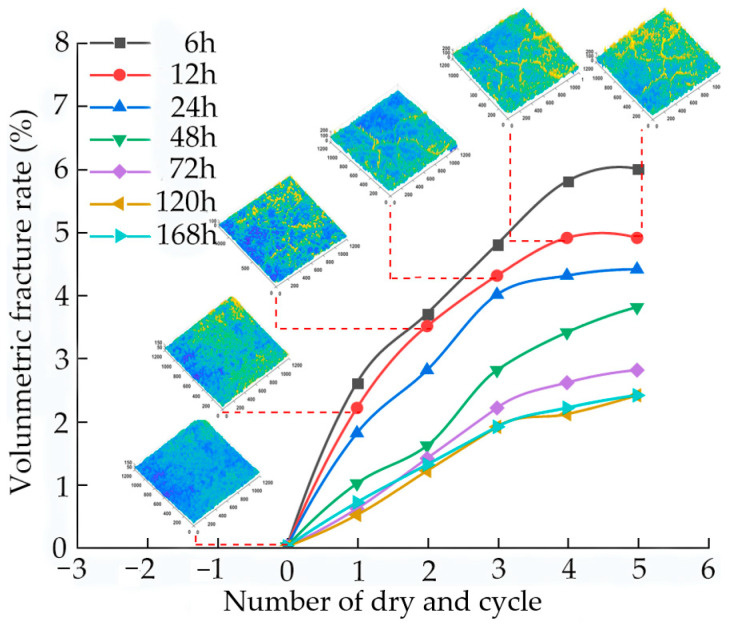
Variation of volume crack rate of sample with dry–wet cycle times and corresponding crack image.

Figure 6 shows the volume crack rate and the corresponding typical cracking images of the samples for different aging times under the dry–wet cycles. During the dry–wet cycle, the volume crack rate increases with increase in the number of dry–wet cycles. The shorter the aging time, the greater the influence of the number of dry–wet cycles on the volume crack rate, which increases first, and then tends to be stable with the additional increase in the aging time. The volume crack rate of the sample aged for 72 h is 53.3% lower than that aged for 6 h. When the aging time is extended to 168 h, the volume crack rate of the sample decreases by 60%, and the overall decreasing trend is not obvious.

### 3.3. Effects of Aging Time on Expansion and Shrinkage of Samples

**Figure 7:**
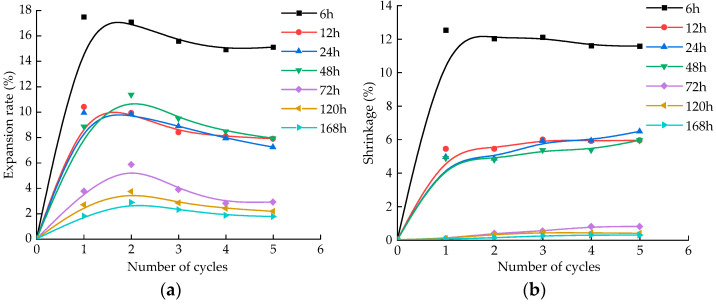
Relationship between aging time, cycle times, and expansion shrinkage: (**a**) relationship between aging time cycle times and expansion rate; (**b**) relationship between aging time cycle times and shrinkage.

Figure 6 shows the relationship between aging time, number of cycles, and expansion shrinkage rate after five dry–wet cycles. The expansion rate of the sample decreases with the increase in aging time (Figure 7a). When the aging time is 72 h, the expansion rate decreases to 4%. If the aging time is prolonged, then the change trend of the expansion rate is not obvious, and the change rate is about 1–2%. The expansion rate of the sample reaches a peak after one or two dry–wet cycles, and then decreases gradually with the increase in the number of dry–wet cycles. During the first dry and wet cycle, lime soil and water undergo a hydration reaction to produce expansion. If the first hydration reaction is insufficient, the second dry and wet cycle soil sample continues to undergo the hydration reaction. As the hydration reaction continues, the sample starts to expand and crack, the internal damage of the soil is serious, and the expansion rate gradually decreases.

The shrinkage of the sample decreases with an increase in aging time. The shrinkage of the sample with an aging time of 6 h is the largest, and is 11.5 times that of the sample aged for 72 h (Figure 7b). With the extension of aging time to 72 h, the shrinkage deformation of the sample tends to be stable. If the aging time is increased, the shrinkage deformation of the sample remains small, and the shrinkage change is about 0–1%. In the first drying and wetting cycle, the lime soil undergoes a hydration reaction and expands. During the drying process, the lime soil shrinks after losing water, and the shrinkage rate of lime soil is large; with the progress of the hydration reaction, the lime soil expands and cracks gradually, the water-holding capacity of lime soil is poor, and the shrinkage rate decreases gradually.

**Figure 8:**
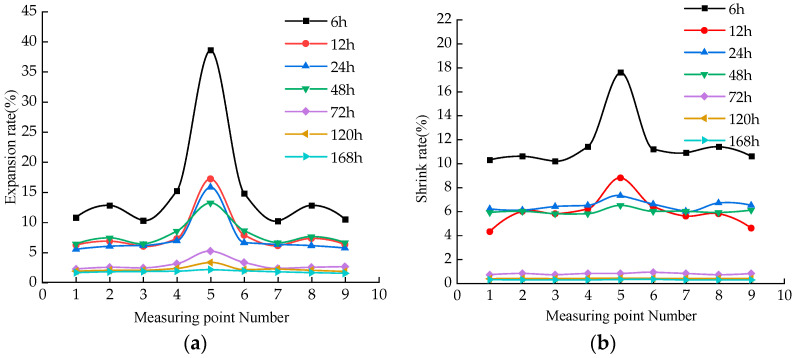
Samples with different aging times at 9 different fulcrums: (**a**) expansion rate; shrink rate; (**b**) shrink rate.

We analyzed the expansion rate of soil samples at different measuring points under the action of dry and wet cycles (Figure 2b), and obtained the data of samples after five dry and wet cycles for processing. Figure 8a shows the expansion rates of samples with different aging times at nine different fulcrums. The expansion rate at the center of the sample is the largest (Number 5 soil sample), the expansion rate at the middle position is the second, and the expansion rate at the corner position is small. When the aging time is 6 h, the expansion rate of the sample at the center is larger, which is 3.78 times of the other minimum expansion rates. With the increase in aging time, the expansion rate of the center of the sample gradually decreases. When the aging time is 72 h, the expansion rate changes at different positions basically tend to be the same. The aging time is short, and due to the influence of hydration reaction, the soil sample bulges at the center, while the surrounding of the soil sample is constrained by the appliance, and the expansion rate is small. With the increase in aging time, the influence of hydration on the sample decreases gradually, and the expansion rate of the whole soil sample decreases.

Figure 8b shows the change law of shrinkage rate of samples at different positions, which is similar to the change law of expansion rate, and the shrinkage rate at the center of the sample is the largest. When the aging time is 6 h, the shrinkage of the sample at the center is the largest. With the increase in aging time, the shrinkage rate at the center of soil sample decreases gradually. When the aging time is 72 h, the reduction in shrinkage rate is large, and the change of shrinkage rate in different positions is gradually consistent. When the aging time is short, the soil sample reaches the maximum value of the expansion rate of the sample when it absorbs water. The internal damage of the soil sample is relatively serious. During the drying process, the soil sample has a large shrinkage potential.

### 3.4. Effects of Aging Time on pH

**Figure 9:**
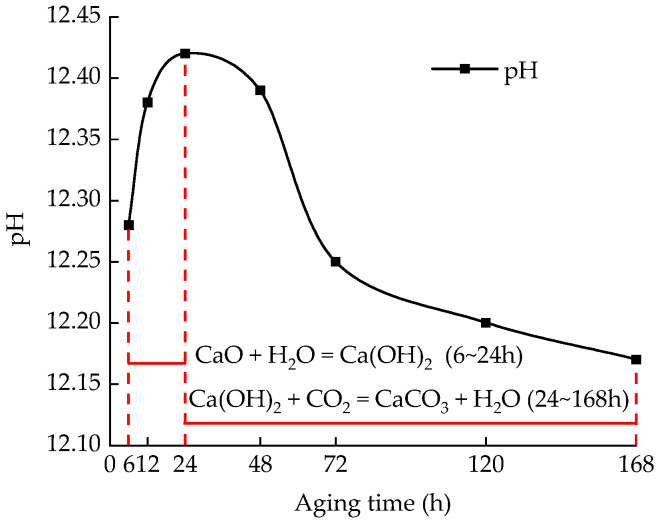
Test results of pH value of samples at different aging time.

The pH value test results are shown in Figure 9. The pH value of the sample increases first, and then decreases with the increase in aging time. When the aging time is 24 h, the pH value of the sample reaches the peak value. At the initial stage of aging, CaO in lime soil rapidly reacts with water to produce a large amount of Ca(OH)_2_ in a short time, resulting in an increase in the pH value of the sample. With the increase in aging time, Ca(OH)_2_ reacts with CO_2_ in the air to form CaCO_3_ crystals, which consumes part of OH^−^ in the sample, and the pH value of the sample decreases. The decreasing rate of the pH value accelerates when the aging time is 72 h, and the decreasing rate is 0.11%. With the further extension of aging time, the content of Ca(OH)_2_ decreases gradually, the carbonation of CO_2_ in the air decreases, and the OH^−^ consumed by CaCO_3_ crystallization is relatively limited, so the reduction rate of the pH value of the sample slows down, and gradually tends to be stable.

### 3.5. Effects of Aging Time on Particle Size

**Figure 10:**
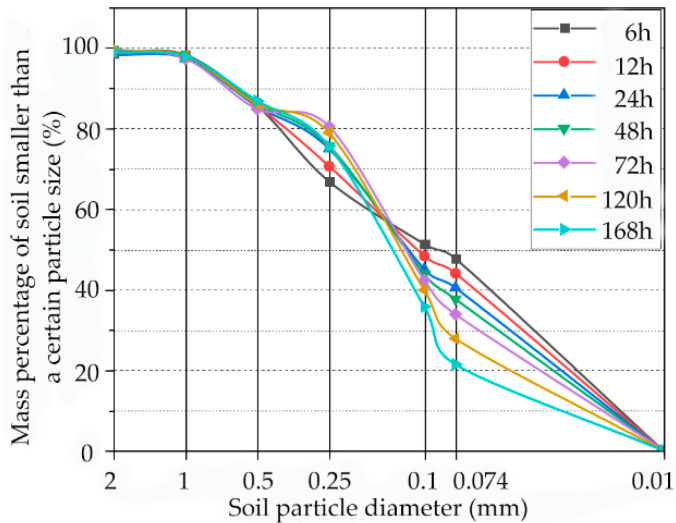
Particle gradation curves of samples with different aging times.

The content of each particle size of samples with different aging times is shown in Figure 10. With the increase in aging time, the content of particles smaller than 0.1 mm decreases, and the content of particles smaller than 0.1–0.5 mm increases. Compared with the sample aged for 72 h, the content of fine particles less than 0.1 mm decreases by 17.5%, and the content of particles of size 0.1–0.5 mm increases by 23.7%. In the aging process, lime and soil particles react chemically, and obvious agglomeration effects occur. Small particles are cemented together to form large particles, and a relatively stable bond is formed between particles [33,34].

### 3.6. Effects of pH on Dry Shrinkage Cracking of Samples

#### 3.6.1. Effects of pH on Volume Crack Rate of Sample

**Figure 11:**
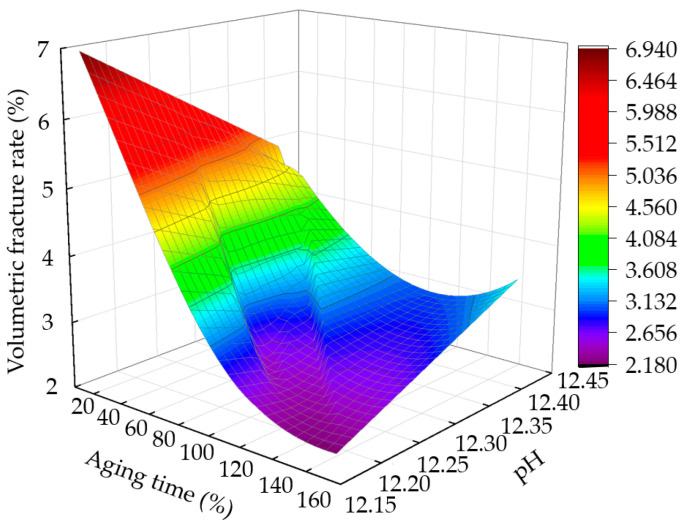
Relationship between volume fracture rate, aging time, and pH value.

**Figure 12:**
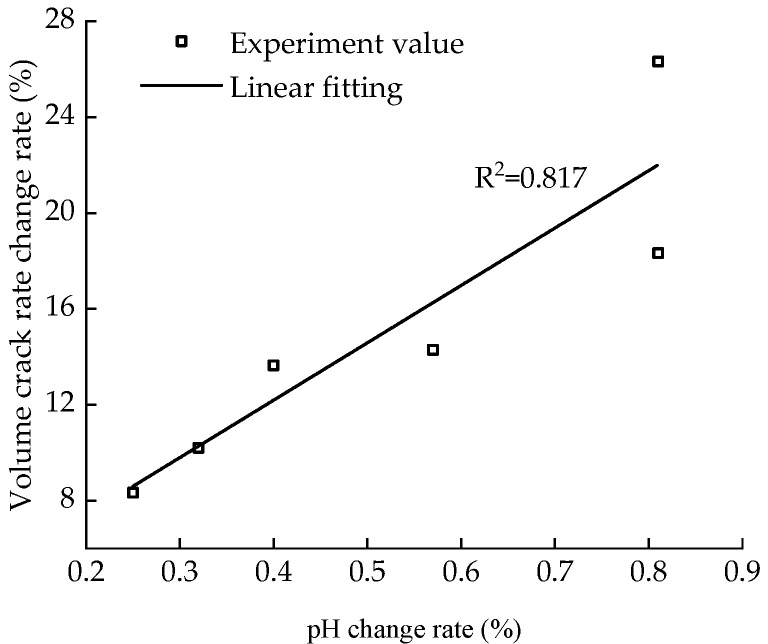
Correlation between pH value change rate and volume fracture rate change rate.

Figure 11 shows the variation curve of the volume crack rate with aging time and pH after five dry–wet cycles. At the initial stage of aging, the pH of the sample is low, and the volume crack rate is high. As the aging time increases, the pH first increases and then decreases, and the volume fracture rate decreases. Figure 12 shows the fitted curve of the correlation between pH change rate and volume fracture rate change; it is observed that there is a good, linear, positive correlation between the volume fracture rate and pH within a certain time, with a correlation value of 0.817. With the increase in pH change rate, the amount of Ca(OH)_2_ produced by the CaO hydration reaction increases, and the volume crack rate resulting from the secondary hydration reaction decreases.

#### 3.6.2. Effects of pH on Sample Expansion

**Figure 13:**
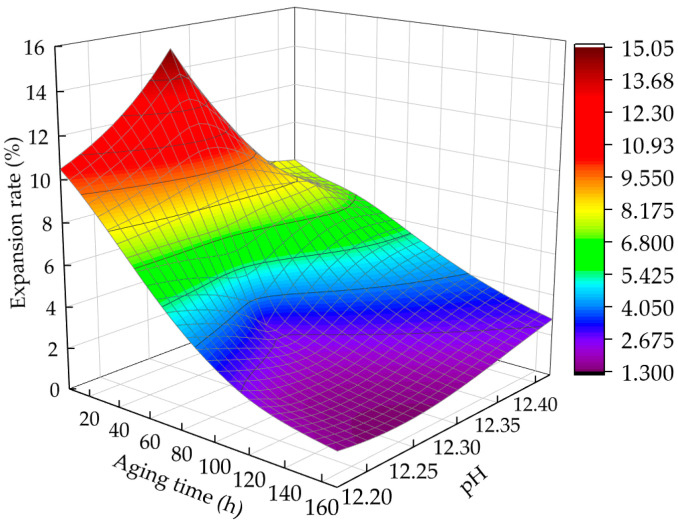
Relationship between expansion rate, aging time, and pH value.

Figure 13 shows the change curve of the expansion rate with aging time and pH after five dry–wet cycles. When the aging time is 6 h, the pH of the sample is small, and the expansion rate is maximal. With the increase in aging time, the pH first increases and then decreases, and the expansion rate continues to decrease.

**Figure 14:**
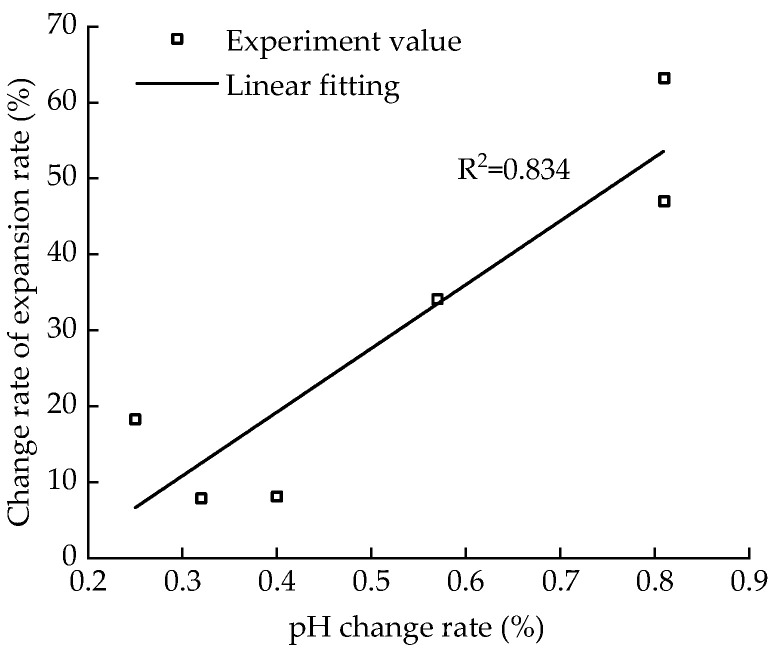
Correlation between pH value change rate and expansion rate change rate.

Figure 14 shows the fitted curve of the correlation between the change rate of the pH value and that of the expansion rate. Given a certain aging time, the correlation between the change rate of the pH and decrease in expansion rate is very high, with a good linear relationship. When the aging time is 6–72 h, the change rate of pH is large, the change rate of Ca(OH)_2_ is accelerated, and the expansion rate is reduced. 

#### 3.6.3. Effects of pH on Sample Shrinkage

**Figure 15:**
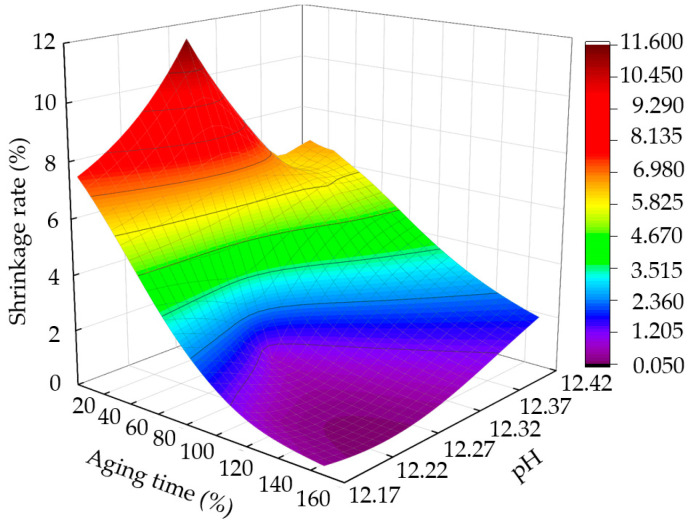
Relationship between shrinkage, aging time, and pH value.

**Figure 16:**
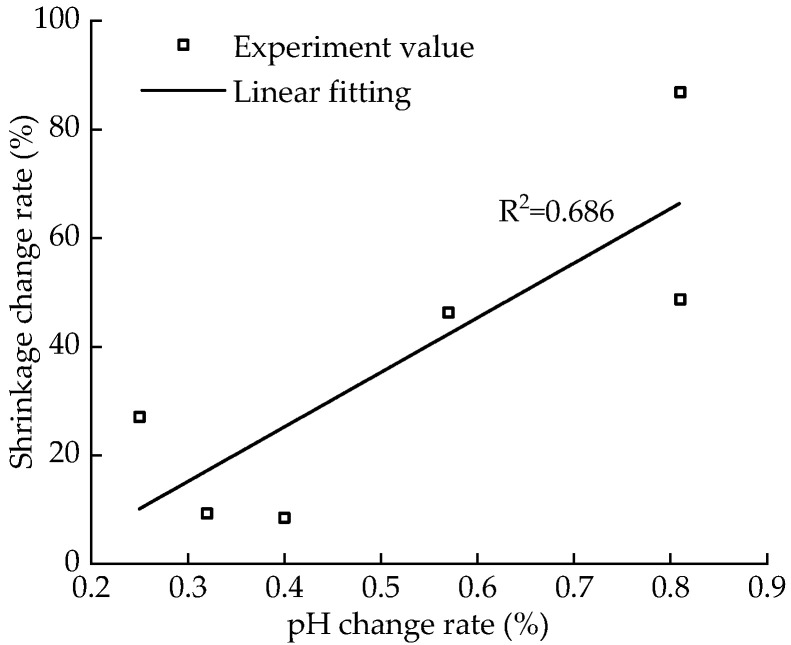
Correlation between pH value change rate and shrinkage change rate.

The shrinkage of the sample reflects its water-holding capacity. Figure 15 shows the change curve for shrinkage with aging time and pH after five dry–wet cycles. With the increase in aging time, the pH first increases and then decreases, and the shrinkage continues to decrease. Figure 16 shows the fitted curve of the correlation between the change rate of pH and that of shrinkage, with a good, linear, positive correlation between the shrinkage and pH for a certain aging time. When the aging time is short, the pH value is low, expansion cracking occurs after the secondary hydration reaction of the sample, the internal damage of the soil is serious, the water-holding capacity of the sample is poor, and the shrinkage rate is large [35,36].

**Figure 17:**
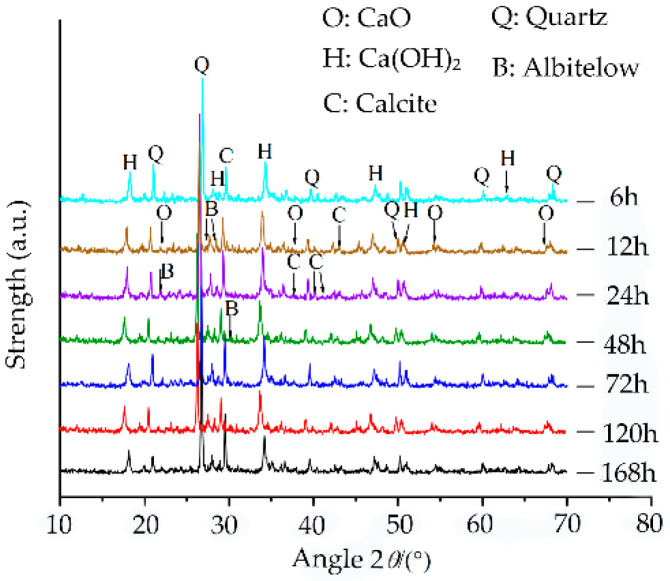
X-ray diffraction test results of samples.

**Figure 18:**
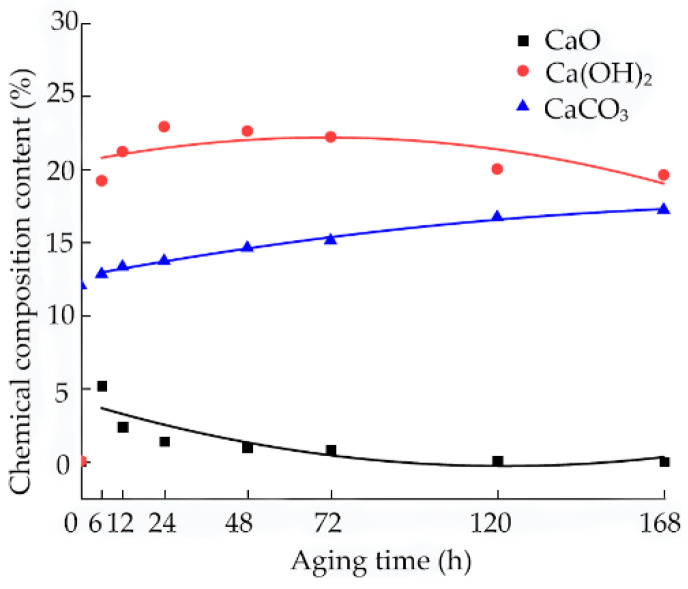
Fitting curve of X-ray diffraction test results.

During aging, the change in the pH value of the sample is closely related to the amount of Ca(OH)_2_ produced by the CaO hydration reaction. To further explore the internal mechanism of the effect of the pH value on lime soil cracking under different aging times, seven sets of samples with different aging times were selected for the XRD test to explore the formation and change law of the internal products of the samples. Figure 17 shows the XRD results, analysis of which shows that lime soil contains CaO, Ca(OH)_2_, CaCO_3_ crystals, and other substances. Quantitative analysis of the XRD results is carried out, and the analysis results are shown in Figure 18. The following results are obtained by analysis:

(1) Figure 15 shows the XRD analysis results of soil samples with different aging times. The changes in material composition of soil samples with different aging times can be obtained by XRD. The peak points of the chemical substances of the soil samples at different aging times change. With the increase in aging time, the peak intensity of CaO decreases, but the peak intensity of Ca(OH)_2_ increases first, and then decreases. The CaO hydration reaction generates Ca(OH)_2,_ which leads to the increase in the peak intensity of Ca(OH)_2_. Affected by carbonization reaction, Ca(OH)_2_ reacts with CO_2_ in the air, so that the peak intensity of Ca(OH)_2_ decreases, and the peak intensity of CaCO_3_ gradually increases. However, the diffraction peak intensity of the CaCO_3_ crystals is low, the content of CO_2_ in natural air is low, the carbonization reaction is slow, and the final carbonization products are limited [37,38];
(2) In the aging process, the content of CaO decreases with the increase in aging time. When the aging time is 6 h, a large amount of the CaO reacts with water quickly to form Ca(OH)_2_, and the content of CaO decreases to 5.2%. When the aging time is extended to 72 h, the content of CaO decreases to 0.8%. If the aging time is extended further, the overall decreasing trend is not obvious, and the content changes remain within 0.05–0.73%;
(3) The content of Ca(OH)_2_ increases first, and then decreases with the extension of the aging time, and a peak value is obtained at 24 h. The change in the Ca(OH)_2_ content tends to remain stable at 72 h of aging, and even after the aging time is prolonged. The overall decrease trend is not obvious, and the change in Ca(OH)_2_ content is about 1.4–1.8%. The change trend of the pH value is consistent with that of Ca(OH)_2_.

### 3.7. Effects of Particle Size on Dry Shrinkage Cracking of Samples

**Figure 19:**
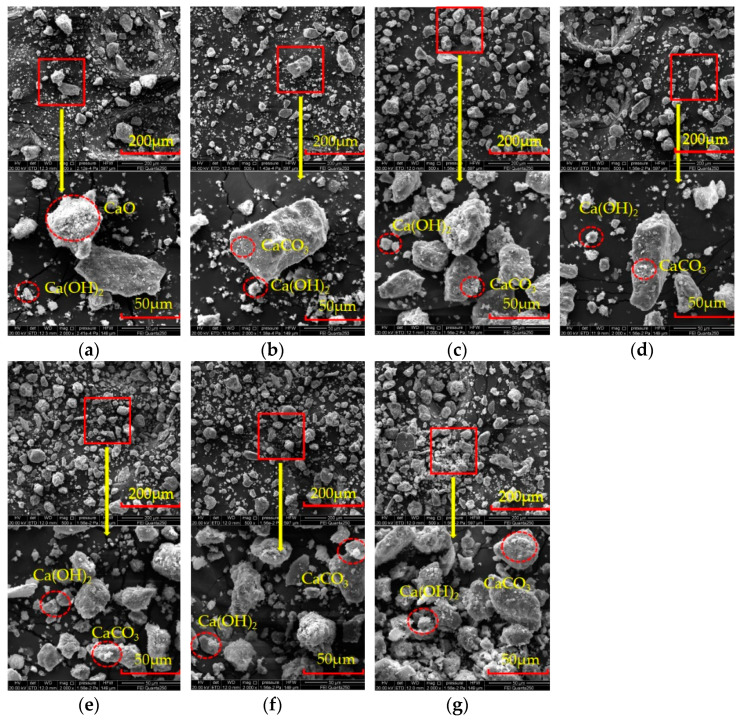
SEM images of samples with different aging times at 500× and 2000× magnification: (**a**) 6 h; (**b**) 12 h; (**c**) 24 h; (**d**) 48 h; (**e**) 72 h; (**f**) 120 h; (**g**) 168 h.

In order to further explore the internal mechanism of the influence of particle size on lime soil cracking, the selected representative samples were tested by 500 times and 2000 times scanning electron microscope. The results are shown in Figure 19. The observation shows that the aging time causes obvious changes in the internal particle structure. When the aging time is 6 h (Figure 19a), the morphology of Ca(OH)_2_ just after the hydration reaction is disordered and irregular, and is prone to stress concentration. From the macroscopic point of view, the soil is easy to crack. With the increase in aging time (Figure 19a–e), the soil particles have an obvious agglomeration effect. When the aging time is 72 h, the particle size and outline of the pellets gradually tend to be stable (Figure 19e).

**Figure 20:**
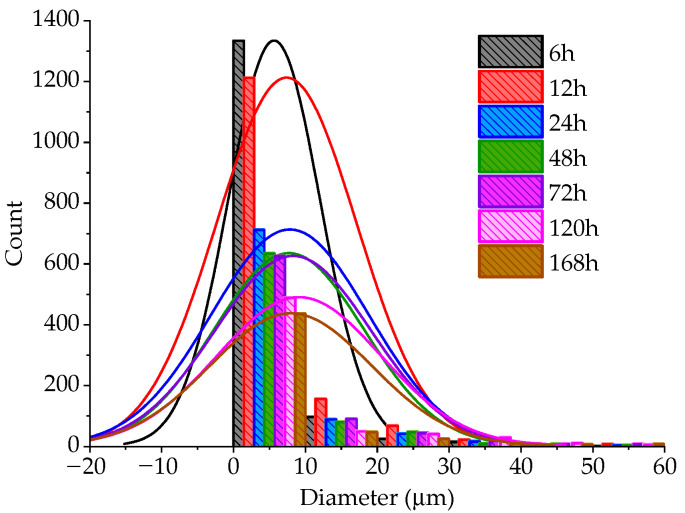
Normal distribution of particle size at different aging times.

We further studied the particle size change of the particles, conducted contrast and denoising on the electron microscope images using Photoshop software, and then measured the particle size using Image Pro Plus 6.0 software to obtain the particle size of the particles in different aging times under the micro state. Figure 20 is a normal distribution diagram of particle size at different aging times.

At the initial stage of aging, the particle size is small; With the increase in aging time, the particle size increases gradually. When the aging time is 72 h, the particle size still increases. Aggregates and clay fine particles are wrapped by calcified crystals and silicates, and small particles are bonded together to form stable, bonded, large particles. An obvious agglomeration effect occurs between particles. After the carbonization reaction, particles only form a carbonized layer on the surface of soil particles, while soil particles are only aggregates of loose particles, which cannot achieve strong cementation. Moreover, due to the carbonization effect, the cohesion between clay particles is partially lost, resulting in the reduction in the shear strength and durability of lime soil.

## 4. Conclusions

Through unconsolidated, undrained triaxial shear tests, dry–wet cycles, screening, pH evaluation, and other tests, the effects of different aging times on the mechanical properties and dry shrinkage cracking of lime soil are explored at the macro scale. Using SEM and XRD tests, the changes in the particle structure and formation of new substances at the micro scale are analyzed. The following observations are obtained from this study:

(1) The increase in aging time is at the expense of soil strength. With the increase in aging time, carbonization and agglomeration reactions occur on the soil surface. However, it is difficult for the agglomerates and particles to form cementation, which leads to the obvious deterioration in the micro mechanical properties and shear strength of the soil sample, including the obvious softening of the stress–strain curve, the serious loss of shear strength, and the attenuation of cohesion;
(2) When the aging time is 6 h, the expansion rate and shrinkage rate at the center of the soil sample are the maximum. With the increase in aging time, the change in expansion and contraction rates at different positions gradually tends to be the same. The volume crack and expansion shrinkage rates first decrease, and then tend to stabilize with the increase in aging time. Compared with a sample aged for 6 h, the volume crack rate of the sample aged for 72 h decreases by 53%, the expansion rate decreases by 14.5%, and the shrinkage rate decreases by 10.5%. With the extension of aging time, the change rates of these three characteristic parameters are about 0–1% each;
(3) The decrease in volume crack rate and expansion shrinkage rate with aging time is positively correlated with the change rate of the pH value, and pH is the internal reason affecting soil cracking. The pH increases first, and then decreases with the increase in aging time. When the sample reaches the reasonable aging time, the pH of the sample shows an obvious decreasing trend. At this time, the changes in Ca(OH)_2_ and CaCO_3_ tend to be stable, the hydration reaction is completed, and the cracking potential is reduced. If the aging time is prolonged, the carbonization reaction causes the shear strength of the soil to decrease, and affects the durability of the soil;
(4) A reasonable aging time improves the particle agglomeration of lime soil. With an increase in aging time, the soil particles undergo obvious agglomeration, the proportion of particles of a size less than 0.1 mm decreases, and the proportion of particles of size 0.1–0.5 mm increases. After an aging time of 72 h, the particle size distribution remains almost unchanged.

## Figures and Tables

**Table 1:** Chemical composition of soil sample.

SiO_2_	Al_2_O_3_	CaO	Fe_2_O_3_	MgO	K_2_O	Na_2_O	TiO_2_	P_2_P_5_	MnO	SO_3_	SrO	ZnO
27.02	8.49	7.52	4.31	1.89	1.13	1.11	0.49	0.32	0.09	0.09	0.02	0.02

**Table 2:** Chemical composition content of quicklime.

CaO	MgO	SO_4_	Fe	Pb	NO_3_	Cl	Unsolvable in Acetic Acid
98.64	0.50	0.10	0.015	0.005	0.004	0.003	0.05

## Data Availability

Not applicable.

## References

[1] Naeimi M., Haddad A. (2020). Environmental impacts of chemical and microbial grouting. Environ. Sci. Pollut. R..

[2] Akula P., Naik S.R., Little D.N. (2021). Evaluating the Durability of Lime-Stabilized Soil Mixtures using Soil Mineralogy and Computational Geochemistry. Transp. Res. Rec. J. Transp. Res. Board.

[3] Yue J.W., Li W.H., Zhu X., Kong Q.M., Huang X.J., Yang X., Han Z.G. (2022). Experimental Study on Restoration Materials of Newly Earthen Ruins under Different Slaking Times. Materials.

[4] Kong D., Chen J., Wan R., Liu H.L. (2020). Study on restoration materials for historical silty earthen Sites based on lime and starch ether. Adv. Mater. Sci. Eng..

[5] Cui Y.J., Tang A.M., Xu M.N. (2011). Effects of the maximum soil aggregates size and cyclic wetting–drying on the stiffness of a lime-treated clayey soil. Géotechnique.

[6] Branco F.C., Belgas M.D., Mendes C., Pereira L., Ortega J.M. (2021). Mechanical Performance of Lime Mortar Coatings for Rehabilitation of Masonry Elements in Old and Historical Buildings. Sustainability.

[7] Yue J.W., Su H.C., Song X., Xu X.C., Zhao L.M., Zhao G., Li P., Chen Y. (2022). Experimental Study on the Cracking and Mechanical Properties of Lime Soil with Different Slaking Conditions of Newly Repaired Earthen City Walls. Materials.

[8] Consoli N.C., Rosa A.D., Saldanha R.B. (2011). Parameters Controlling Strength of Industrial Waste-Lime Amended Soil. Soils Found.

[9] Zukri A. (2013). Pekan Soft Clay Treated with Hydrated Lime as a Method of Soil Stabilizer. Procedia Eng..

[10] Olinic T., Olinic E. (2016). The Effect of Quicklime Stabilization on Soil Properties. Agric. Agric. Sci. Procedia.

[11] Poncelet N., Herrier G., Franois B. (2021). An effective stress constitutive framework for the prediction of desiccation crack in lime-treated soil: Experimental characterization and constitutive prediction. Geomech. Energy Environ..

[12] Stoltz G., Cuisinier O., Masrouri F. (2012). Multi-scale analysis of the swelling and shrinkage of a lime-treated expansive clayey soil. Appl. Clay Sci..

[13] Zhang S.B., Xie J.B., Jiang S., Shi L., Wang S., Wu Y.C. (2018). Research on the development law of dry shrinkage cracks in lime improved expansive soil. Contrib. Technol..

[14] Elert K., Rodriguez N.C., Pardo E.S. (2002). Lime mortars for the conservation of historic buildings. Stud. Conserv..

[15] Grilo J., Santos S.A., Faria P. (2014). Mechanical and mineralogical properties of natural hydraulic lime metakaolin mortars in different curing conditions. Constr. Build. Mater..

[16] Gu L., Liu R.J., Guo Z.W. (2012). Effect of lime digestion conditions on calcium hydroxide activity. China Powder Sci. Technol..

[17] Mascolo G., Mascolo M.C., Vitale A., Marino O. (2010). Microstructure evolution of lime putty upon aging. J. Cryst. Growth.

[18] Wei G.F., Zhang B.J., Fang S.Q. (2012). Aging Mechanism of Quicklime and Application Study of Aged Lime in Conservation of Cultural Relics. J. Bulid. Mater..

[19] Khattab S.A.A., Hussein Y.A. (2012). On the Durability of fine Grained Soils Stabilized With Lime. Al-Rafadain Eng. J..

[20] Hengique H.M., Santos L.C., Parreira P.M. (2010). Production of milk of lime for sugar cane industry: Study of factors influencing lime slaking. Mater. Sci. Forum.

[21] Sweeney D.A., Wong D.K.H., Fredlund D.G. (1998). Effect of lime on highly plastic clay with special emphasis on aging. Transp. Res. Rec..

[22] Tan Y.Z., Yu B., Zheng A., Fu W., Zhang H., Wan Z. (2013). Long-term carbonated effect on strength of lime stabilized laterite soils. J. Bulid. Mater..

[23] Cui K., Feng F., Chen W.W., Wang X.H., Cheng F.Q. (2019). Study on the mechanical compatibility of fissure grouting slurry with quick lime and grouting technology optimization in earthen sites. Rock Soil Mech..

[24] Maafi N., Akchiche M., Sara R. Wetting and Drying Compacted Soil-Lime Mixtures. Proceedings of the International Congress and Exhibition “Sustainable Civil Infrastructures: Innovative Infrastructure Geotechnology”.

[25] Ouhadi V. (2016). Microstructural Assessment of Lime Consumption Rate and Pozzolanic Reaction Progress of a Lime-Stabilized Dispersive Soil. Modares Civ. Eng. J..

[26] Yue J., Chen Y., Zhao L., Wang S., Su H., Yang X., Gao H., Zhang Y., Li W. (2021). Effects of Aging on the Dry Shrinkage Cracking of Lime Soils with Different Proportions. Appl. Sci..

[27] Feng H.P., Ma D.L., Liu Q.Y., Ye C.L. (2013). Method for calculating three dimensional apparent porosity of soils based on SEM images. J. Bulid. Mater..

[28] Guidobaldi G., Cambi C., Cecconi M., Deneele D., Paris M., Russo G., Vitale E. (2017). Multi-scale analysis of the mechanical improvement induced by lime addition on a pyroclastic soil. Eng. Geol..

[29] Pakbaz M.S., Farzi M. (2014). Comparison of the effect of mixing methods (dry vs. wet) on mechanical and hydraulic properties of treated soil with cement or lime. Appl. Clay Sci..

[30] Li X.M., Lu G.Y., Zhang H.Y., Yi S., Ren K.B. (2021). Strength characteristics and micro-mechanism of lime-Metakaolin Modified silty Soil. J. Bulid. Mater..

[31] Bhuvaneshwari S., Robinson R.G., Gandhi S.R. (2014). Behaviour of Lime Treated Cured Expansive Soil Composites. Indian Geotech. J..

[32] Yang Y., Zhang N., Zhang L., Rong H., Wei C.J. (2021). Influence of Modified Materials on Hygrothermal properties of Raw Soil Blocks. J. Bulid. Mater..

[33] Arizzi A., Viles H., Cultrone G. (2012). Experimental testing of the durability of lime based mortars used for rendering historic buildings. Constr. Build. Mater..

[34] Wang D.X., He F.J., Zhu J.Y. (2019). Performance and mechanism of CO_2_ carbonated slag-CaO-MgO-solidified soils. Acta Geotech..

[35] Wang Y., Cui Y.J., Tang A.M., Tang C.S., Benahmed N. (2015). Effects of aggregate size on water retention capacity and microstructure of lime-treated silty soil. Géotech. Lett..

[36] Margalha M.G., Silva A.S., Maria D.R.V., Jorge D.B., James B.R., Charles A.G. (2013). Microstructural Changes of Lime Putty during Aging. J. Mater. Civ. Eng..

[37] Muzahim A.M., Khattb S., Alcover J.F. (2012). Microstructure and geotechnical properties of lime-treated expansive clayey soil. Eng. Geol..

[38] Mateos M. (2015). Soil Lime Research at Iowa State University. Gypsum. Lime.

